# Dynamic survival prediction using longitudinal AGR in unresectable locally advanced esophageal squamous cell carcinoma

**DOI:** 10.3389/fimmu.2026.1909638

**Published:** 2026-08-31

**Authors:** Feng Du, Wei Deng, Yanjie Xiao, Rong Yu, Jun Jia

**Affiliations:** 1 Key Laboratory of Carcinogenesis and Translational Research (Ministry of Education/Beijing), The VIPII Gastrointestinal Cancer Division of Medical Department, Peking University Cancer Hospital and Institute, Beijing, China; 2 Key Laboratory of Carcinogenesis and Translational Research (Ministry of Education/Beijing), Department of Radiation Oncology, Peking University Cancer Hospital & Institute, Beijing, China

**Keywords:** albumin-to-globulin ratio, dynamic prediction, esophageal squamous cell carcinoma, immune checkpoint inhibitor, prognosis

## Abstract

**Background:**

Immune checkpoint inhibitor (ICI)-based induction therapy followed by definitive radiotherapy has emerged as a promising treatment strategy for patients with unresectable locally advanced esophageal squamous cell carcinoma (LA-ESCC). However, many patients still experience disease progression and poor clinical outcomes. Although longitudinal circulating biomarkers may provide valuable prognostic information, their dynamic predictive value in this treatment setting remains largely unexplored.

**Materials and methods:**

We retrospectively enrolled patients with unresectable LA-ESCC from a single medical center who received PD-1inhibitor-based induction therapy followed by definitive radiotherapy. In addition to baseline clinical and histopathological characteristics, 110 circulating biomarkers were serially collected throughout treatment to characterize longitudinal changes. A Bayesian joint model integrating baseline predictors with longitudinal biomarker trajectories was developed for dynamic survival prediction. Model discrimination and calibration were evaluated using time-dependent area under the receiver operating characteristic curve (AUCs) and Brier scores, respectively.

**Results:**

A total of 376 patients were included, comprising a training cohort (n = 221) and an independent temporal test cohort (n = 155), yielding 328,104 longitudinal biomarker measurements. Following multistage variable selection procedure, latent class growth analysis identified distinct longitudinal trajectories of the albumin-to-globulin ratio (AGR) that are significantly associated with survival outcomes. Dynamic AGR value and trajectory emerged as the most informative longitudinal predictors among all candidate biomarkers. In the training cohort, the 12-, 24-, and 36-month AUCs were 0.85 (95% CI, 0.82-0.89), 0.92 (95% CI, 0.90-0.94), and 0.87 (95% CI, 0.84-0.91), respectively. In the test cohort, the corresponding 12- and 24-month AUCs were 0.80 (95% CI, 0.75-0.85) and 0.82 (95% CI, 0.78-0.86), respectively.

**Conclusion:**

By integrating longitudinal AGR trajectories with baseline clinical information, the Bayesian joint model enables individualized, real-time prognostic updating and may facilitate dynamic risk stratification and risk-adapted management for patients with unresectable LA-ESCC.

## Introduction

Esophageal squamous cell carcinoma (ESCC) imposes a substantial global health burden, particularly in East Asia (1, 2). For patients with unresectable locally advanced ESCC (LA-ESCC), definitive chemoradiotherapy remain the cornerstone of treatment, while the addition of immunotherapy has recently emerged as a promising strategy to further improve outcomes (3–7). However, therapeutic efficacy remains limited by considerable interpatient heterogeneity (4, 8, 9). The inability to reliably identify high-risk patients early in the stage course hinders the implementation of personalized, risk-adapted treatment strategies, underscoring the urgent need for more accurate prognostic models.

Treatment failure after radiotherapy results from a complex interplay of clinical, pathological, and immunological factors (10–13). Circulating biomarkers provide a minimally invasive and readily repeatable approach for monitoring real-time systemic and tumor-associated changes during treatment (14–17). Among these, the albumin-to-globulin ratio (AGR) reflect both systemic inflammation and nutritional status, two key determinants of response to chemoradiotherapy and immunotherapy (18, 19). Accumulating evidence has established AGR as a robust and reproducible prognostic biomarker across multiple malignancies. A recent meta-analysis demonstrated that a higher pretreatment AGR was significantly associated with improved overall survival (OS) in patients receiving immune checkpoint inhibitors (ICIs) (18).The prognostic value of AGR has also been validated in resettable ESCC, where a high preoperative AGR is associated with improved 5-year survival and better postoperative quality of life (20), whereas a low AGR predicts poorer clinical outcomes (21). Collectively, these findings support AGR as a simple, inexpensive, and reproducible prognostic biomarker in ESCC across different treatment settings.

Nevertheless, most prognostic models incorporating circulating biomarkers remain static. Models based on single baseline measurements fail to capture the temporal changes in biomarkers and their evolving relationship with clinical outcomes (22, 23). In contrast, dynamic prediction models continuously update risk estimates by incorporating longitudinal follow-up data (24, 25). Statistical approaches such as latent class growth analysis (26), and Bayesian joint modeling enable longitudinal biomarker trajectories to be linked with survival outcomes, thereby extracting richer prognostic information from serial measurements (27).

To address these limitations, we enrolled patients with LA-ESCC who received PD-1 inhibitor-based induction therapy followed by definitive chemoradiotherapy and collected baseline clinical characteristics together with serial circulating biomarkers throughout treatment. A data-driven screening strategy was applied to a comprehensive panel of longitudinal biomarkers, leading to the identification of AGR as the most informative prognostic biomarker. We then integrated longitudinal AGR dynamic trajectories with baseline clinical characteristics using a Bayesian joint model to develop a dynamic prognostic framework capable of continuously updating individualized risk estimates, thereby addressing the current lack of longitudinal biomarker-based prediction models for patients with unresectable LA-ESCC.

## Methods

### Participants

This retrospective study consecutively enrolled patients with unresectable LA-ESCC who were treated at Peking University Cancer Hospital from January 2018 through December 2025. Eligible patients were aged ≥ 18 years and had histologically confirmed ESCC. Patients with supraclavicular lymph node metastases were eligible, whereas those with distant metastasis to other organs were excluded. All patients received one to four cycles of PD-1 inhibitor-based induction therapy followed by definitive radiotherapy.

Baseline hematologic parameters were obtained from laboratory test results performed within 30 days before treatment initiation, and at least one baseline measurement was required for study inclusion. Patients were excluded if they had a concurrent second primary malignancy of non-esophageal origin or lacked essential baseline clinical data.

For model development, patients treated from 2018 to 2022 comprised the training cohort. The prediction model was developed using this cohort and internally validated through bootstrap resampling. Patients treated from 2023 to 2025 constituted an independent temporal test cohort, which was used to evaluate the model’s predictive performance and temporal generalizability.

### Outcomes

The primary endpoint was progression-free survival (PFS), defined as the time from treatment initiation to the first documented disease progression or death from any cause. Patients who were alive and progression-free at the last follow-up were censored on the date of their last follow-up.

### Predictors and feature processing

Candidate predictors included baseline clinical and pathological characteristics together with serial blood-based biomarkers measured longitudinally from treatment initiation until the end of follow-up. The biomarker panel comprised 110 parameters, including hematologic indices, and organ function tests, nutritional and metabolic markers, inflammatory biomarkers, coagulation parameters, and tumor-associated markers.

Missing data were handled using multiple imputation, which was performed separately for the training and test cohorts. Continuous variables were retained on their original scale or log-transformed, as appropriate, to improve distributional normality.

### Model development

#### Baseline Cox survival model construction

Repeated measurements obtained within the baseline assessment window were averaged to generate baseline values. Variables with more than 30% missing data were excluded before imputation. Missing values in the remaining variables were inputted using multiple imputation. Least absolute shrinkage and selection operator (LASSO)-penalized Cox regression was used for prognostic feature selection, followed by stepwise model selection based on the Akaike information criterion (AIC). A multi-variable Cox proportional hazards model was then constructed using the screened predictors.

#### Longitudinal joint model development

##### Data processing

Only longitudinal blood biomarker measurements obtained within the first 24 months after treatment initiation were included in the longitudinal analyses because measurements beyond this time point became increasingly sparse. These measurements were aligned into consecutive 30-day intervals beginning at baseline. When multiple measurements of the same biomarker were available within a given interval, they were averaged to generate a single representative value for that interval.

#### Feature selection

##### Step 1

Missing data filtering. For each marker, the proportion of missing values was calculated across all longitudinal measurements obtained during the first 24 months of follow-up. The average missing rate over this period was then determined. Biomarkers with an average missing rate ≤30% were retained for further analysis.

##### Step 2

Longitudinal trajectory selection. For each retained marker, linear mixed-effects models were fitted to characterize longitudinal trajectories, and the best optimal functional form was selected using the Akaike information criterion (AIC). Biomarkers with P values below the Bonferroni-adjusted significance threshold were retained for the next step.

##### Step 3

Univariate joint modeling screening. For each biomarker retained after Step 2, a separate univariate Bayesian joint model was fitted by combining the selected longitudinal sub-model with baseline Cox survival model. Biomarkers with a posterior P-value ≤ 0.05 were considered prognostically informative and were retained for subsequent model development.

#### Latent class trajectory modeling

The retained longitudinal biomarkers were analyzed using latent class growth mixture models with random intercepts to identify distinct patient subgroups based on their longitudinal biomarker trajectories. The optimal number of latent classes was determined by jointly considering the AIC, Bayesian Information Criterion (BIC), sample-size-adjusted BIC (SABIC), entropy, the requirement that each class comprised >2% of the study population, statistical significance (P < 0.05), and clinical interpretability. Model selection was performed iteratively by progressively increasing the number of latent classes.

### Joint model specification and estimation

A Bayesian joint modeling framework was implemented using the JMbayes2 package in R. This framework jointly models longitudinal biomarker trajectories and survival outcomes by linking the previously developed baseline Cox model with linear mixed-effects models for longitudinal biomarkers. Specifically, the joint model comprised: (1) a linear mixed-effects sub-model for the selected longitudinal biomarkers; (2) latent class membership incorporated into the longitudinal sub-model as a time-varying effect modifier to capture heterogeneous biomarker trajectories across patient subgroups; and (3) a survival sub-model formulated as a Cox proportional hazards model incorporating baseline prognostic factors. The current value of the estimated longitudinal biomarker trajectory, m_i(t), was linked to the survival submodel via an association parameter α, corresponding to the current value association structure (**Supplementary Table 3**).

The overall sequential feature-selection workflow is illustrated in **Supplementary Figure 1**. Detailed screening results for all candidate biomarkers at each stage of the selection process are summarized in **Supplementary Table 1**, and additional methodological details are described in **Supplementary Methods S1**.

### Model evaluation

Model performance was evaluated in terms of discrimination and calibration using time-dependent AUCs and the Brier score, respectively. Internal validation was performed in the training cohort, whereas temporal external validation were conducted in the independent test cohort. Bootstrap resampling (50 replicates) was used to assess model robustness and estimate 95% confidence intervals for performance metrics.

### Statistical analysis

Statistical analyses were performed using R software (version 4.4.2). Categorical variables are presented as frequencies and percentages and were compared between groups using the χ^2^ test or Fisher’s exact test, as appropriate. Continuous variables are presented as mean (SD) ± standard deviation (SD) or median with interquartile range (IQR), as appropriate, and were compared using Student’s t-test or the Mann-Whitney U test. All statistical tests were two-sided, and statistical significance was defined as P < 0.05.

This study was conducted and reported in accordance with the Strengthening the Reporting of Observational Studies in Epidemiology (STROBE) guideline (28) and the Transparent Reporting of a Multivariable Prediction Model for Individual Prognosis or Diagnosis-Artificial Intelligence (TRIPOD-AI) guideline (29).

### Ethics statement

The studies involving human participants were reviewed and approved by the Medical Ethics Committee of Beijing Cancer Hospital (Approval No. 2022KT41, March 9, 2022). All participants provided written informed consent before enrollment. This study was registered in the Medical Research Registration Information System (Registration No. MR-11-22-003459).

## Results

### Baseline characteristics

A total of 456 patients were initially screened, of whom 376 met the inclusion criteria and were enrolled in the study. The study population comprised 221 patients in the training cohort (2018-2022) and 155 in the independent temporal test cohort (2023-2025). Baseline characteristics are summarized in **Table 1**. The overall cohort had a mean age of 62.9 years (SD, 7.77), was predominantly male (89%), and mainly presented with locally advanced disease, including T3 tumors (57%), and N2 nodal stage (50%). All patients with M1 disease (41%) had distant metastases confined to the supraclavicular lymph nodes.

**Table 1:** Baseline characteristics of the training and test cohorts.

Feature	Overall (n = 376)	Training cohort (n = 221)	Test cohort (n = 155)
Age	62.9 (7.77)	63.4 (7.90)	62.3 (7.57)
Missing	6	0	6
Gender			
Male	329 (89%)	197 (89%)	132 (89%)
Female	41 (11%)	24 (11%)	17 (11%)
Missing	6	0	6
BMI	22.5 (3.25)	22.5 (3.28)	22.4 (3.21)
Missing	7	1	6
T stage			
T1-T2	34 (9.3%)	22 (10.0%)	12 (8.2%)
T3	211 (57.5%)	112 (51.0%)	99 (67.8%)
T4	122 (33.2%)	87 (39.0%)	35 (24.0%)
Missing	9	0	9
N stage			
N0	10 (2.8%)	5 (2.3%)	5 (3.5%)
N1	88 (24%)	47 (21%)	41 (28%)
N2	183 (50%)	113 (52%)	70 (49%)
N3	82 (23%)	54 (25%)	28 (19%)
Missing	13	2	11
M stage			
M0	221 (59%)	143 (65%)	78 (50%)
M1	155 (41%)	78 (35%)	77 (50%)
Missing	0	0	0
Tumor location			
Cervical	60 (16%)	35 (16%)	25 (17%)
Upper	81 (22%)	46 (21%)	35 (24%)
Middle	123 (34%)	77 (35%)	46 (31%)
Lower	84 (23%)	44 (20%)	40 (27%)
Multiple lesions	19 (5.2%)	18 (8.2%)	1 (0.7%)
Missing	9	1	8
Tumor grade			
Carcinoma in situ	6 (1.8%)	0 (0%)	6 (4.5%)
Well	11 (3.2%)	3 (1.4%)	8 (6.1%)
Moderately	181 (53.2%)	118 (57%)	63 (47.7%)
Poorly	142 (41.8%)	87 (42%)	55 (41.7%)
Missing	36	13	23
PD-L1 status			
CPS=0	36 (19%)	26 (22%)	10 (15%)
CPS≥1	149 (81%)	92 (78%)	57 (85%)
Missing	191	103	88

BMI, body mass index; CPS, combined positive score; PD-L1, programmed death-ligand 1. Data are presented as mean (SD) for continuous variables and n (%) for categorical variables.

Longitudinal data included 110 blood-based biomarkers (**Supplementary Table 1**). In total, 328,104 biomarker measurements were collected. Patients in the training cohort had a mean of 10.7 longitudinal assessments (SD 6.7), compared with 7.3 assessments (SD 3.7) in the test cohort.

### Baseline survival model development

Following application of the predefined baseline assessment window and exclusion of variables with a high proportion of missing data, 30 candidate predictors were retained for analysis. LASSO-penalized Cox regression was used for initial feature selection followed by bidirectional stepwise regression based on the minimum AIC criterion. Three predictors including metastatic stage, cytokeratin 19 fragment (CYFRA 21-1), and aspartate aminotransferase (AST) were ultimately retained in the baseline multivariable Cox proportional hazards model (**Supplementary Table 2**).

### Construction of the longitudinal joint model

#### Longitudinal feature selection

After excluding biomarkers with a high proportion of missing data, 16 candidate biomarkers were retained for longitudinal analysis. Linear mixed-effects models were used to characterize the longitudinal trajectories of each biomarker, and the optimal functional form of time was selected based on the lowest AIC/BIC. Each longitudinal sub-model was subsequently integrated with the baseline Cox proportional hazards model within a Bayesian joint modeling framework. Biomarkers were retained if they demonstrated a significant association with progression-free survival, achieved stable model convergence, and were clinically interpretable. Ultimately, three biomarkers including AGR, albumin, and indirect bilirubin were retained for the final joint model.

#### Latent class trajectory modeling

**Figure 1:**
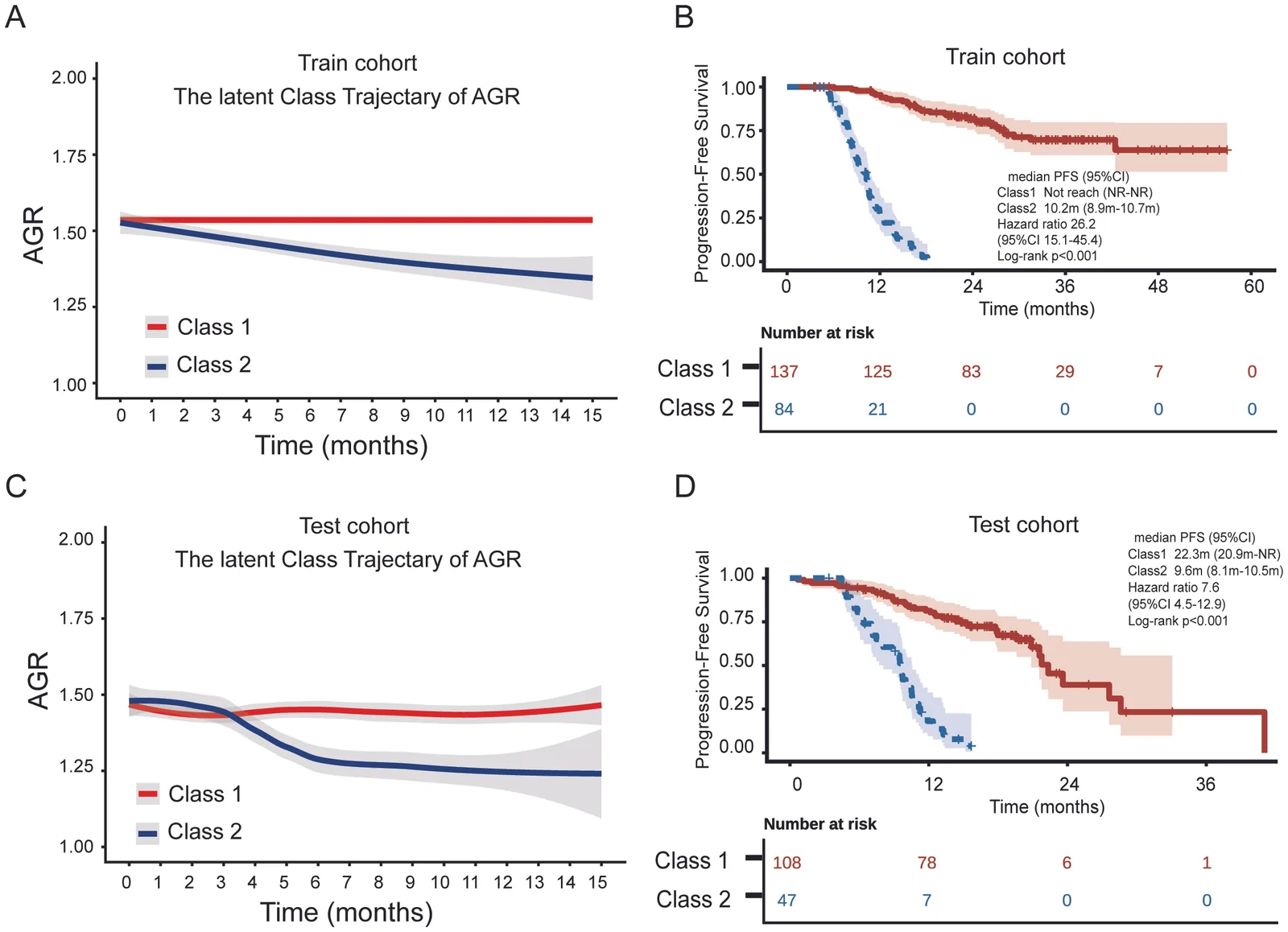
Latent trajectory classes of albumin-to-globulin ratio (AGR) and their association with progression-free survival (PFS). Longitudinal AGR trajectories of the two latent classes in the **(A)** training and **(C)** temporal test cohorts. Solid lines represent the estimated mean AGR trajectories, and shaded areas indicate the corresponding 95% confidence intervals (CIs). Kaplan-Meier curves for PFS stratified by AGR trajectory class are shown for the **(B)** training cohort and **(D)** temporal test cohorts. Shaded pink and light-blue areas represent the 95% CIs for survival estimates of Class 1 and Class 2, respectively. Hazard ratios (HRs) with 95% CIs are shown in each survival panel; NR indicates that the PFS was not reached. Numbers at risk are displayed below each Kaplan-Meier survival curve.

Latent class trajectory analysis was performed independently for each of the three selected biomarkers. For AGR, a two-class model provided the optimal fit, classifying 38% and 62% of patients into two distinct trajectory classes. Class 1 maintained a relatively stable AGR trajectory throughout follow-up, whereas Class 2 exhibited a marked decline during the first few months after treatment initiation (**Figure 1A**).

In contrast, no distinct longitudinal trajectory patterns were identified for albumin or indirect bilirubin. Despite having comparable baseline characteristics (**Table 2**), patients in Class 2 experienced significantly shorter PFS than those in Class 1 (**Figure 1B**), highlighting the prognostic significance of longitudinal AGR trajectory patterns.

**Table 2:** Baseline characteristics of two albumin-to-globulin ratio latent classes in the training cohort.

Feature	Class 1 (n = 137)	Class 2 (n= 84)	*P* value
Age	64 (57, 69)	62 (58, 69)	> 0.9
Gender			0.7
Male	123 (89.8%)	74 (88.1%)	
Female	14 (10.2%)	10 (11.9%)	
T stage			0.5
T2	16 (11.7%)	6 (7.1%)	
T3	69 (50.3%)	43 (51.2%)	
T4	52 (38.0%)	35 (41.7%)	
N stage			0.5
N0-N1	32 (23.4%)	18 (21.4%)	
N2	72 (52.6%)	41 (48.8%)	
N3	31 (22.6%)	25 (29.8%)	
Missing	2(1.5%)	0(0.0%)	
M stage			0.013
M1	41 (29.9%)	39 (46.4%)	
M0	96 (70.1%)	45 (53.6%)	
Tumor location			0.2
Cervical	25 (18.2%)	10 (11.9%)	
Upper	29 (21.2%)	17 (20.2%)	
Middle	51 (37.2%)	26 (31.0%)	
Lower	21 (15.3%)	23 (27.4%)	
Multiple lesions	11 (8.1%)	7 (8.3%)	
Missing	0	1(1.2%)	
PD-L1			0.5
CPS = 0	17 (12.4%)	9 (10.7%)	
CPS ≥ 1	54 (39.4%)	38 (45.2%)	
Missing	66(48.2%)	37(44.1%)	

CPS, combined positive score; PD-L1, programmed death-ligand 1. Data are presented as median (Q1, Q3) for continuous variables and n (%) for categorical variables.

P values were calculated using the Wilcoxon rank-sum test for continuous variables and the chi-square test or Fisher's exact test for categorical variables, as appropriate.

#### Joint longitudinal survival model

A Bayesian joint longitudinal-survival model was subsequently developed by integrating three components: (1) a linear mixed-effects sub-model for the longitudinal AGR trajectory, (2) the corresponding latent class as an effect modifier, and (3) a baseline Cox proportional hazards sub-model. Models incorporating one, two, or three longitudinal biomarkers were systematically compared. The model including AGR alone was selected as the final model because it achieved the lowest deviance information criterion (DIC) and highest log pseudo-marginal likelihood (LPML), indicating the best balance between model fit and parsimony (**Table 3**). Accordingly, the AGR-based joint model was retained for subsequent validation (**Supplementary Table 3**). 

**Table 3:** Information criterion-based comparison of Bayesian joint models with different longitudinal biomarker combinations.

Longitudinal feature included in model	DIC	WAIC	LPML
AGR only	119.6	120.77	-76.19
AGR + indirect bilirubin	1,637.43	1,667.26	-872.66
indirect bilirubin only	2,499.54	2,497.05	-1,248.80
AGR + ALB	11,385.76	11,460.40	-5,839.59
ALB only	12,328.94	12,325.38	-6,163.89
AGR + ALB + indirect bilirubin	12,876.26	12,958.61	-6,547.84
ALB + indirect bilirubin	13,824.46	13,836.24	-6,923.56

AGR, albumin-to-globulin ratio; ALB, albumin; DIC, deviance information criterion; LPML, log pseudo-marginal likelihood; WAIC, Watanabe-Akaike information criterion.

**Figure 2:**
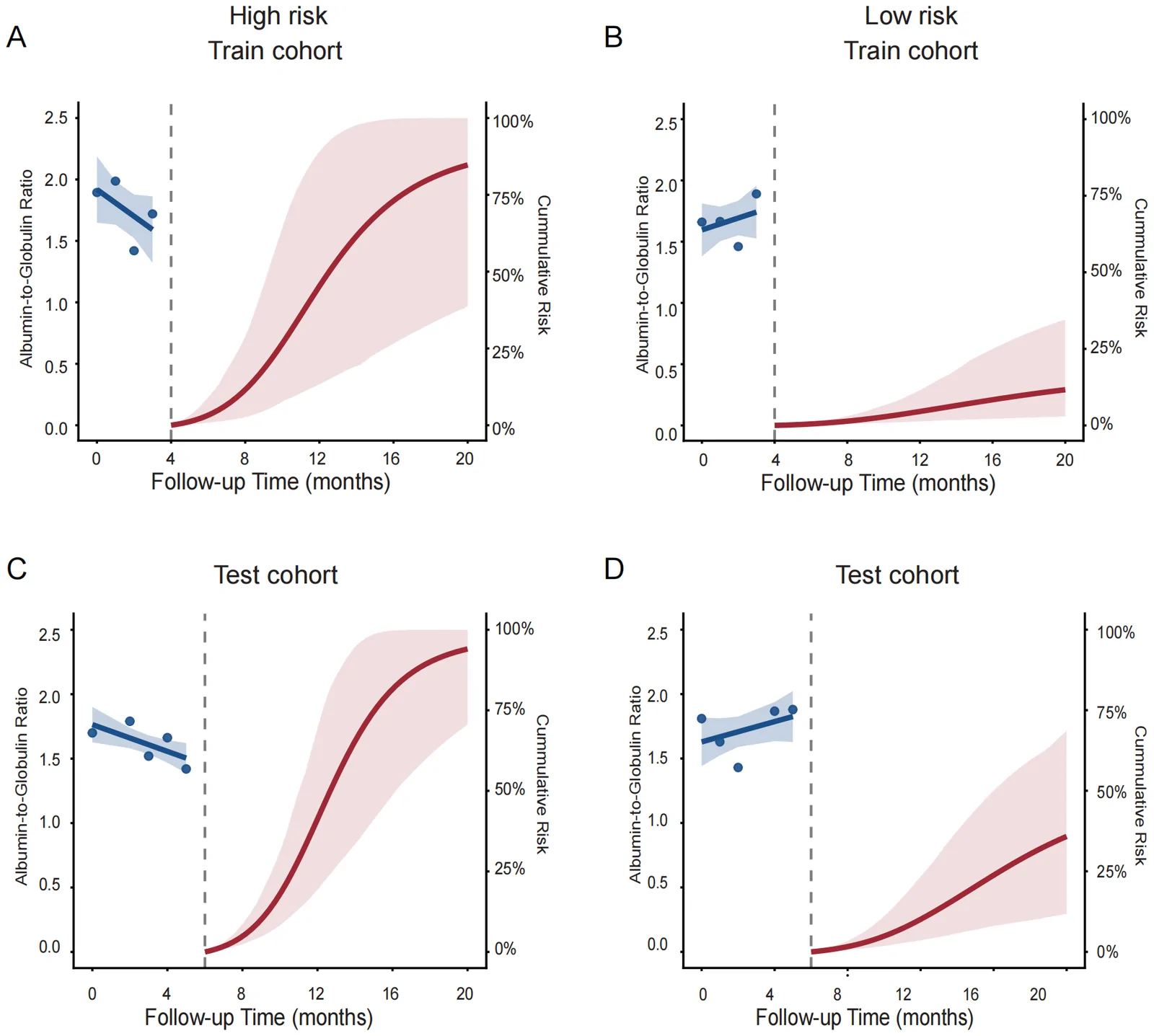
Individualized dynamic prediction of progression risk based on longitudinal AGR trajectories using the Bayesian joint model. Representative examples are shown for a high-risk patient from the **(A)** training cohort, a low-risk patient from the **(B)** training cohort, a high-risk patient from the **(C)** temporal test cohort, and a low-risk patient from the **(D)** temporal test cohort. Each panel is divided by a vertical gray dashed line into an observation period (left) and a prediction period (right). During the observation period, blue circles represent serial AGR measurements, the blue solid line denotes the fitted longitudinal trajectory, and the light-blue shaded area indicates the corresponding 95% confidence interval (CI). During the prediction period, the red solid line represents the model-predicted cumulative risk of disease progression based on the observed AGR trajectory, with the light-red shaded area indicating the corresponding 95% CI.

The final Bayesian joint longitudinal-survival model was fitted using the jm() function in the R package JMbayes2. The prespecified Cox proportional hazards model served as the survival submodel, whereas the linear mixed-effects model for longitudinal AGR measurements served as the longitudinal submodel. The longitudinal and survival processes were linked through a current-value association structure, whereby the instantaneous hazard of progression or death was associated with the estimated current value of the underlying subject-specific AGR trajectory at the corresponding time point. As illustrated in **Figure 2**, patients with a stable AGR trajectory maintained a consistently low cumulative risk of disease progression, whereas those with a rapid early decline in AGR exhibited a marked increase in cumulative progression.

### Model validation and performance assessment

#### Internal validation

**Figure 3:**
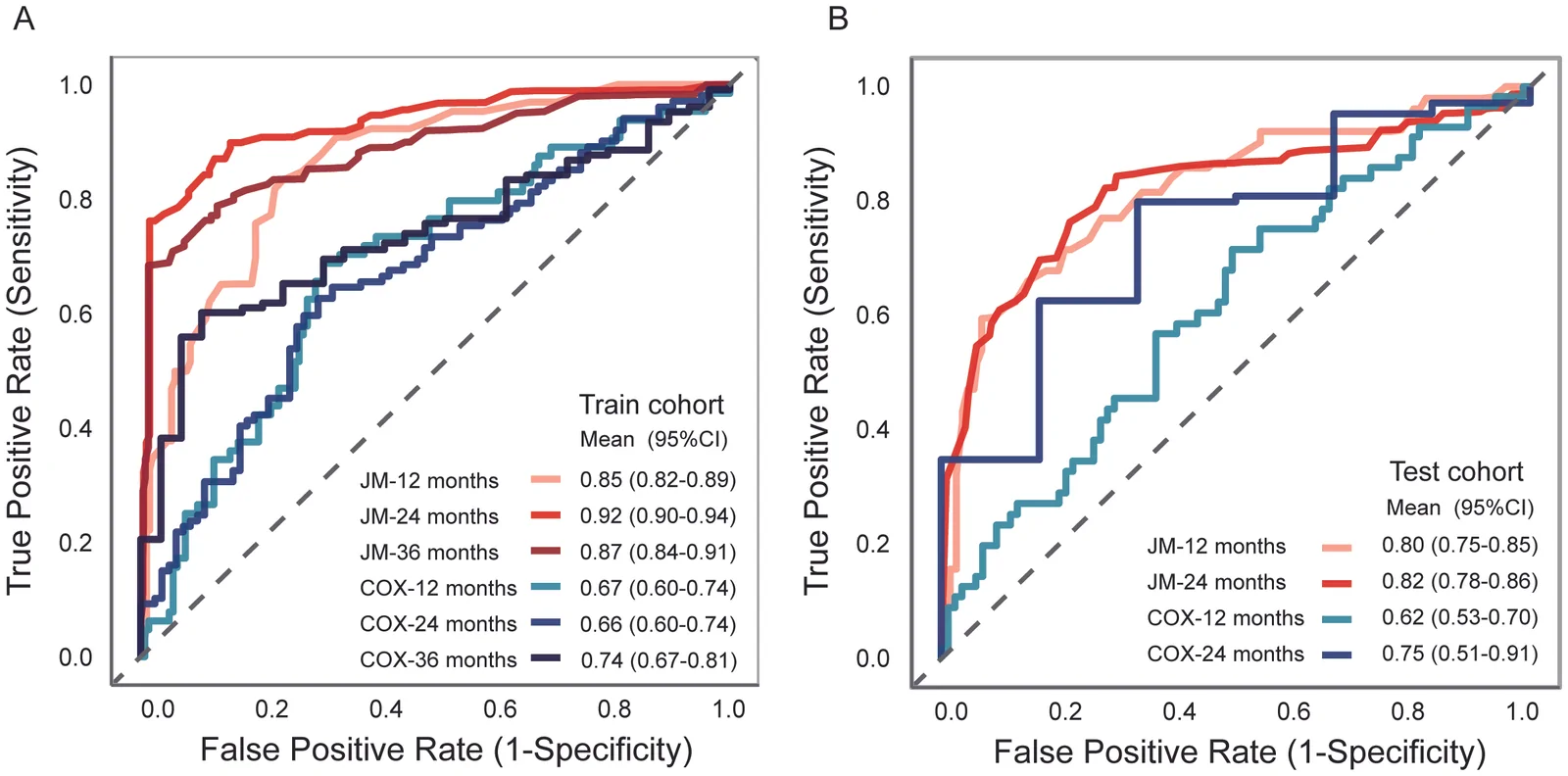
Time-dependent receiver operating characteristic (ROC) analysis comparing the Bayesian joint model (JM) and the baseline Cox regression model in the training and test cohorts. **(A)** Time-dependent ROC curves and corresponding area under the curve (AUCs) with 95% confidence intervals (CIs) at 12, 24, and 36 months in the training cohort. **(B)** Time-dependent ROC curves and the corresponding AUCs with 95% CIs at 12 and 24 months in the temporal test cohort. Higher AUCs values indicate better discrimination between patients with and without disease progression.

Internal validation using 50 bootstrap replicates demonstrated robust model performance. The time-dependent AUCs were 0.85 (95% CI, 0.82-0.89), 0.92 (95% CI, 0.90-0.94), and 0.87 (95% CI, 0.84-0.91) at 12, 24, and 36 months, respectively (**Figure 3A**). Calibration was also excellent, with corresponding Brier scores of 0.09 (95% CI, 0.07-0.11), 0.13 (95% CI, 0.12-0.15), and 0.12 (95% CI, 0.10-0.13). Compared with the baseline Cox proportional hazards model, the Bayesian joint model consistently demonstrated superior discrimination across all evaluated time points (**Figure 3A**).

#### External validation

In the independent temporal test cohort, AGR similarly exhibited two distinct longitudinal trajectory patterns that were associated with significantly different PFS outcomes (**Figures 1C, D**). The Bayesian joint model consistently yielded higher time-dependent AUCs than the baseline Cox proportional hazards model across the evaluated time points. The time-dependent AUCs were 0.80 (95% CI, 0.75-0.85) and 0.82 (95% CI, 0.78-0.86) at 12 and 24 months, respectively (**Figure 3B**). Calibration remained good, with Brier scores of 0.11 (95% CI, 0.09-0.13) and 0.15 (95% CI, 0.13-0.17) at 12 and 24 months, respectively.

### Sensitivity analysis

**Figure 4:**
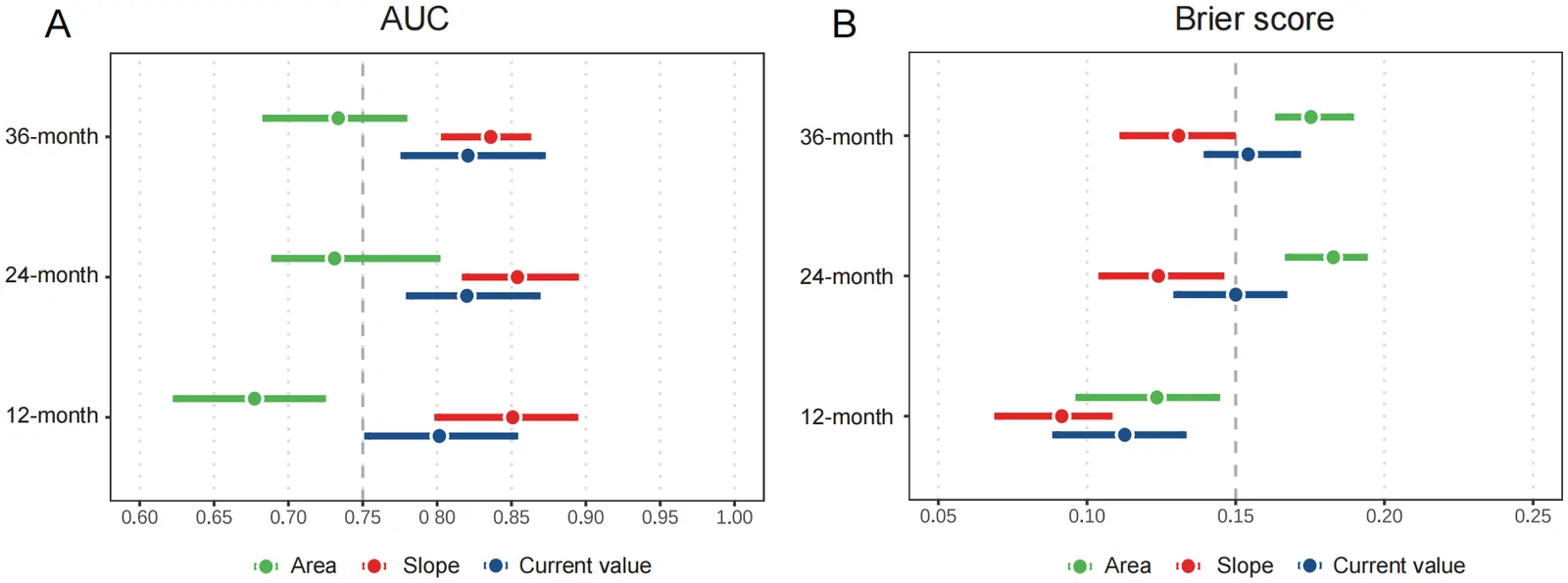
Predictive performance of Bayesian joint models incorporating different longitudinal parameterizations of the albumin-to-globulin ratio (AGR). **(A)** Time-dependent areas under the receiver operating characteristic curve (AUCs) with 95% confidence intervals (CIs) at 12, 24, and 36 month for joint models incorporating cumulative AGR area (green), AGR slope (red), or the current AGR value (blue). **(B)** Corresponding time-dependent Brier scores with 95% CIs at 12, 24, and 36 months for the three AGR-based joint models.

We evaluated the impact of alternative longitudinal AGR metrics on model performance. The slope-based model demonstrated performance comparable to that of the current-value model, with time-dependent AUCs ranging from 0.84 to 0.85 and Brier scores ranging from 0.09 to 0.13 across the evaluated time points. In contrast, the cumulative area-based model showed poorer discrimination and calibration, with AUCs ranging from 0.68 to 0.73 (**Figures 4A, B**).

We further evaluated model performance according to PD-L1 expression status in the pooled training and test cohorts. Comparable AUCs and Brier scores were observed across subgroups, suggesting that model performance was not materially influenced by PD-L1 expression status (**Supplementary Figure 2**).

## Discussion

This study developed a dynamic prediction model based on longitudinal circulating biomarkers to assess progression risk in patients with locally advanced ESCC patients undergoing immunotherapy-based induction treatment followed by definitive chemoradiotherapy. By incorporating longitudinal biomarker trajectories together with baseline clinical characteristics, the model demonstrated improved predictive performance over a conventional baseline Cox model, facilitating earlier identification of patients at increased risk of disease progression.

This study has several strengths. First, we established a dynamic prognostic modeling framework that continuously incorporates several biomarker measurements obtained during treatment, enabling characterization of disease evolution beyond static baseline assessments. Second, systematic trajectory analysis demonstrated that longitudinal AGR dynamics provide independent prognostic information, identifying two distinct trajectory patterns with robust discriminative ability for PFS. Third, the study is supported by a rigorous analytical framework that combined Bayesian joint modeling with latent class trajectory analysis and leveraged systematically collected longitudinal data from a large clinical cohort.

While the prognostic value of baseline AGR has been well-established across multiple solid tumors (18, 30–32), its clinical application has largely relied on a single baseline measurement, offering only a static assessment that does not reflect treatment-related physiological changes. Increasing evidence suggested that longitudinal changes in circulating biomarker provide additional prognostic information beyond baseline measurements (16, 33). A recent pan-cancer study demonstrated that serial peri-operative AGR monitoring improved prognostic stratification beyond baseline values; however, this observation was limited to patients undergoing surgical treatment (34).

The present study extends these findings to patients with unresectable locally advanced ESCC; a clinically heterogeneous population in whom baseline assessments alone may not adequately distinguish subsequent clinical outcomes. Conventional Cox proportional hazards models rely primarily on baseline covariates and therefore cannot incorporate longitudinal changes in biomarkers during treatment. Consequently, they provide only a static estimate of prognosis and may fail to capture dynamic changes in patient risk over time.

In contrast, the Bayesian joint model simultaneously incorporates baseline clinical characteristics and longitudinal biomarker trajectories, allowing individualized risk estimates to be updated as serial laboratory measurements become available. By integrating longitudinal information with survival data, the joint modeling approach provided improved predictive performance compared with the baseline Cox model in the present study. This dynamic updating mechanism enables individualized risk prediction to evolve as new biomarker measurements become available, thereby providing clinically more informative prognostic assessment than a static baseline model.

The prognostic value of longitudinal AGR trajectories may be explained by the composite biological characteristics of this biomarker. Calculated as the serum albumin-to-globulin ratio, AGR simultaneously reflects systemic nutritional status and chronic inflammation activity. A decline in albumin may indicate treatment-related nutritional depletion resulting from esophageal mucosal injury and reduced oral intake (35, 36), whereas an increase in globulin may reflect tumor-associated and radiotherapy-induced inflammatory activation (37, 38).

Within the treatment setting, these processes may collectively impair antitumor immunity. Malnutrition can compromise lymphocyte function and reduce the efficacy of PD-1 blockade (39–41), whereas persistent inflammation may promote radio resistance and an immunosuppressive tumor microenvironment (42, 43). Accordingly, a rapidly declining AGR trajectory may reflect the combined effects of nutritional deterioration and heightened systemic inflammation, whereas a stable AGR trajectory may indicate preserved physiological homeostasis and a more favorable treatment response. Nevertheless, these mechanistic interpretations remain speculative and require further experimental validation, as immune function, inflammatory pathways and mechanisms of radiotherapy resistance were not directly evaluated in the present study.

This dynamic risk stratification framework has potential clinical utility. For patients exhibiting an early decline in AGR, intensified nutritional support and optimization of supportive care may help potentially improve treatment tolerance, although whether such interventions translate into improved oncologic outcomes require prospective evaluation. In addition, these patients may benefit from closer radiologic surveillance to facilitate earlier detection of disease progression and timely salvage treatment, whereas patients with stable AGR trajectories may be appropriately managed using standard follow-up schedules. Importantly, this approach relies on routinely collected laboratory parameters without requiring additional invasive procedures or substantial costs, supporting its potential integration into routine clinical practice.

This study has several limitations. Firstly, all participants were recruited from a single medical center in China, which may limit the generalizability of our findings. External validation in multicenter cohorts with broader geographic and demographic representation is warranted. Second, biomarkers with a high proportion of missing data were excluded from model development, potentially precluding assessment of their prognostic value. Third, the lower predictive performance observed in the temporal validation cohort may reflect both model optimism resulting from internal model development and clinical heterogeneity between the training and validation cohorts. Differences in baseline characteristics, event rates, follow-up duration, longitudinal laboratory measurement frequency, and evolving treatment strategies may all have contributed to reduced external discrimination. Although bootstrap resampling provides an estimate of internal model stability and reduces the impact of model optimism, it cannot fully account for optimism or generalization error in independent populations. Despite these limitations, the Bayesian joint model consistently demonstrated higher discriminative performance than the baseline Cox model in both the training and temporal validation cohorts. Moreover, the reproducible association between longitudinal AGR trajectories and PFS across both cohorts supports the potential robustness of the proposed modeling strategy. Prospective validation in independent multicenter cohorts will be essential before routine clinical implementation.

## Conclusion

This study developed and temporally validated a dynamic prediction model that integrates longitudinal AGR trajectories with baseline clinical characteristics in patients with locally advanced ESCC undergoing immunotherapy-based induction therapy followed by definitive chemoradiotherapy. The model provided individualized, dynamically updated estimates of progression risk and demonstrated improved predictive performance compared with a baseline Cox model. These findings support the potential value of incorporating longitudinal biomarker dynamics into risk stratification and may facilitate more personalized follow-up and clinical decision-making.

## Data Availability

The raw data supporting the conclusions of this article will be made available by the authors, without undue reservation.
