# Supplementary material for: Dynamic survival prediction using longitudinal AGR in unresectable locally advanced esophageal squamous cell carcinoma
Source: Front Immunol. 2026 Aug 31;17:1909638. doi: 10.3389/fimmu.2026.1909638 (PMC13572146; doi:10.3389/fimmu.2026.1909638)
Supplement: Supplementary file 1 [file DataSheet1.pdf]

Supplementary Appendix

Dynamic survival prediction using longitudinal AGR in unresectable locally advanced esophageal squamous cell carcinoma

Table S1. Full list of 110 laboratory parameters considered as candidate predictors/features

Category	Items
**Hematologic Tests (n = 26)**	Mean Corpuscular Hemoglobin Concentration (MCHC), Hematocrit (HCT), Basophil Count (Absolute), Large Platelet Ratio (P-LCR), Basophil Percentage (BASO%), Eosinophil Count (Absolute), Monocyte Count (Absolute), Platelet Distribution Width (PDW), Neutrophil Percentage (NEUT%), Mean Platelet Volume (MPV), Monocyte Percentage (MONO%), Lymphocyte Percentage (LYMPH%), Mean Corpuscular Volume (MCV), Hemoglobin (HGB), White Blood Cell Count (WBC), Mean Corpuscular Hemoglobin (MCH), Lymphocyte Count (Absolute), Red Cell Distribution Width (SD) (RDW-SD), Platelet Count (PLT), Red Cell Distribution Width (CV) (RDW-CV), Red Blood Cell Count (RBC), Eosinophil Percentage (EOS%), Neutrophil Count (Absolute), Erythrocyte Sedimentation Rate (ESR),Reticulocyte Percentage,Reticulocyte Absolute Count
**Organ Function (n = 20)**	Total Bile Acids (TBA), Direct Bilirubin (DBIL), Aspartate Aminotransferase (AST), Indirect Bilirubin (IBIL), Cholinesterase (CHE), Gamma-Glutamyl Transferase (GGT), Alkaline Phosphatase (ALP), Lactate Dehydrogenase (LDH), Alpha-L-Fucosidase (AFU), Globulin (GLOB), Alanine Aminotransferase (ALT), Uric Acid (UA), Creatinine (CREA), Cystatin C (Cys-C), Total Protein (TP), Serum Amylase (AMS), Lipase (LPS), β2-Microglobulin (β2-MG), Myoglobin (MYO), N-terminal pro-B-type Natriuretic Peptide (NT-proBNP)
**Nutritional & Metabolic Markers (n = 18)**	Apolipoprotein B (ApoB), Total Cholesterol (TC), High-Density Lipoprotein Cholesterol (HDL-C), Low-Density Lipoprotein Cholesterol (LDL-C), Triglycerides (TG), Lipoprotein(a) [Lp(a)], Glucose (GLU), Glycated Serum Albumin (GSA), Glycated Hemoglobin (HbA1c), Albumin (ALB), Prealbumin (PA), Albumin-to-Globulin Ratio (AGR), Total Carbon Dioxide (TCO2), Phosphorus (P), Calcium (Ca), Blood Ammonia (NH3), Creatine Kinase (CK), Homocysteine
**Inflammatory & Immune Profiles (n = 14)**	C-Reactive Protein (CRP), Serum Amyloid A (SAA), High-Sensitivity CRP (hs-CRP), Interleukin-10 (IL-10), Interleukin-17A (IL-17A), Interleukin-2 (IL-2), Interleukin-8 (IL-8), Interleukin-1β (IL-1β), Interleukin-6 (IL-6), Interleukin-4 (IL-4), Procalcitonin (PCT), Total B Lymphocyte Percentage, Total T Lymphocyte Percentage, CD8+ T Cell Percentage
**Coagulation Parameters (n = 10)**	Fibrinogen (FIB), Prothrombin Time Ratio (PTR), Activated Partial Thromboplastin Time (APTT), Thrombin Time (TT), Prothrombin Time (PT), Partial Thromboplastin Time (PTT), Plasma Prothrombin Time, Fibrin, D-Dimer (D-D), Fibrin Degradation Products (FDP)
**Tumor-associated Markers (n = 10)**	Neuron-Specific Enolase (NSE), Cytokeratin 19 Fragment (CYFRA 21-1), Carcinoembryonic Antigen (CEA), Squamous Cell Carcinoma Antigen (SCCA), Cancer Antigen 125 (CA125), Pro-Gastrin-Releasing Peptide (ProGRP), Cancer Antigen 242 (CA242), Prostate-Specific Antigen (PSA),

	Free Prostate-Specific Antigen (fPSA), Alpha-Fetoprotein (AFP)
**Endocrine Hormones (n = 12)**	Thyroid-Stimulating Hormone (TSH), Thyroid Hormone (TH), Free Triiodothyronine (FT3), Free Thyroxine (FT4), Triiodothyronine (T3), Thyroxine (T4), Anti-Thyroid Peroxidase Antibody (TPOAb), Thyrotropin Receptor Antibody (TRAb), Thyroglobulin (Tg), Parathyroid Hormone (PTH), Calcitonin (CT), Anti-Thyroglobulin Antibody (TgAb)

Table S2. Baseline Multi-variable Cox Proportional Hazards Models

Feature	HR	95% CI	p value	C-index
Metastatic stage	1.95	1.32-2.86	<0.001	0.640
Cyfra21-1	1.02	1.00-1.05	0.034	
AST	1.02	1.00-1.04	0.060	

Abbreviations: AST, aspartate aminotransferase; CI, confidence interval; Cyfra21-1, cytokeratin 19 fragment antigen 21-1; HR, hazard ratio.

Supplementary Methods S1

Longitudinal biomarker screening and joint model development

Step 1: Missing data filtering. For each marker, the missing rate was calculated at each post-baseline time point (from baseline to 24 months), and the average missing rate across all time points was then derived. Markers with a mean missing rate⩽30% were retained, yielding 16 eligible markers: creatinine, uric acid, albumin-to-globulin ratio, total protein, albumin, total bile acids, direct bilirubin, indirect bilirubin, glucose, aspartate aminotransferase, alanine aminotransferase, lactate dehydrogenase, alkaline phosphatase, phosphorus, gamma-glutamyl transferase, and total carbon dioxide.

Step 2: Longitudinal trajectory selection. For each of the 16 retained markers, linear mixed- effects models were fitted with time as the fixed effect, sequentially incorporating linear, quadratic, and restricted cubic spline terms. The optimal time-effect specification for each marker was chosen based on the Akaike Information Criterion. To address multiple testing, P values for time effects were calculated; markers with P values ≤ the Bonferroni-adjusted threshold (0.05/16 = 0.003)were defined as showing significant temporal variation. This step identified eight markers with dynamic trajectories: phosphorus, direct bilirubin, albumin-to-globulin ratio, indirect bilirubin, creatinine, total protein, albumin, and glucose.

Step 3: Univariate joint modeling screening. For each of the eight markers identified in Step 2, a separate univariate Bayesian joint model was fitted, combining the longitudinal submodel with a survival submodel. Markers with a posterior P-value ⩽0.05 for the association parameter linking the longitudinal trajectory to survival were considered prognostically relevant. Three markers met this criterion: indirect bilirubin, albumin-to-globulin ratio, and albumin.

Step 4: Final model development. Based on the three markers identified in Step 3, Bayesian joint models with all combinations of the selected markers (single-marker, pairwise, and three-marker specifications) were systematically compared. To capture heterogeneity in longitudinal trajectories across patient subgroups, latent class trajectories were first derived for each marker using growth mixture modeling, and the resulting class assignments were incorporated into the longitudinal submodel along with a class-by-time interaction term. All Bayesian joint models were fitted via the JMbayes2 package in R and estimated using Markov chain Monte Carlo (MCMC) sampling. Four parallel MCMC chains were run for each model with 30,000 iterations; the first 10,000 iterations were discarded as burn-in, and every 10 iteration was thinned to mitigate autocorrelation (n_iter = 30000, n_burnin = 10000, n_thin = 10). The optimal specification was selected based on the Deviance Information Criterion (DIC), Watanabe-Akaike Information Criterion (WAIC), and log pseudo-marginal likelihood (LPML), prioritizing both goodness-of-fit and parsimony.

Figure S1. Flowchart illustrating the analytical workflow for longitudinal biomarker screening and Bayesian joint model development.

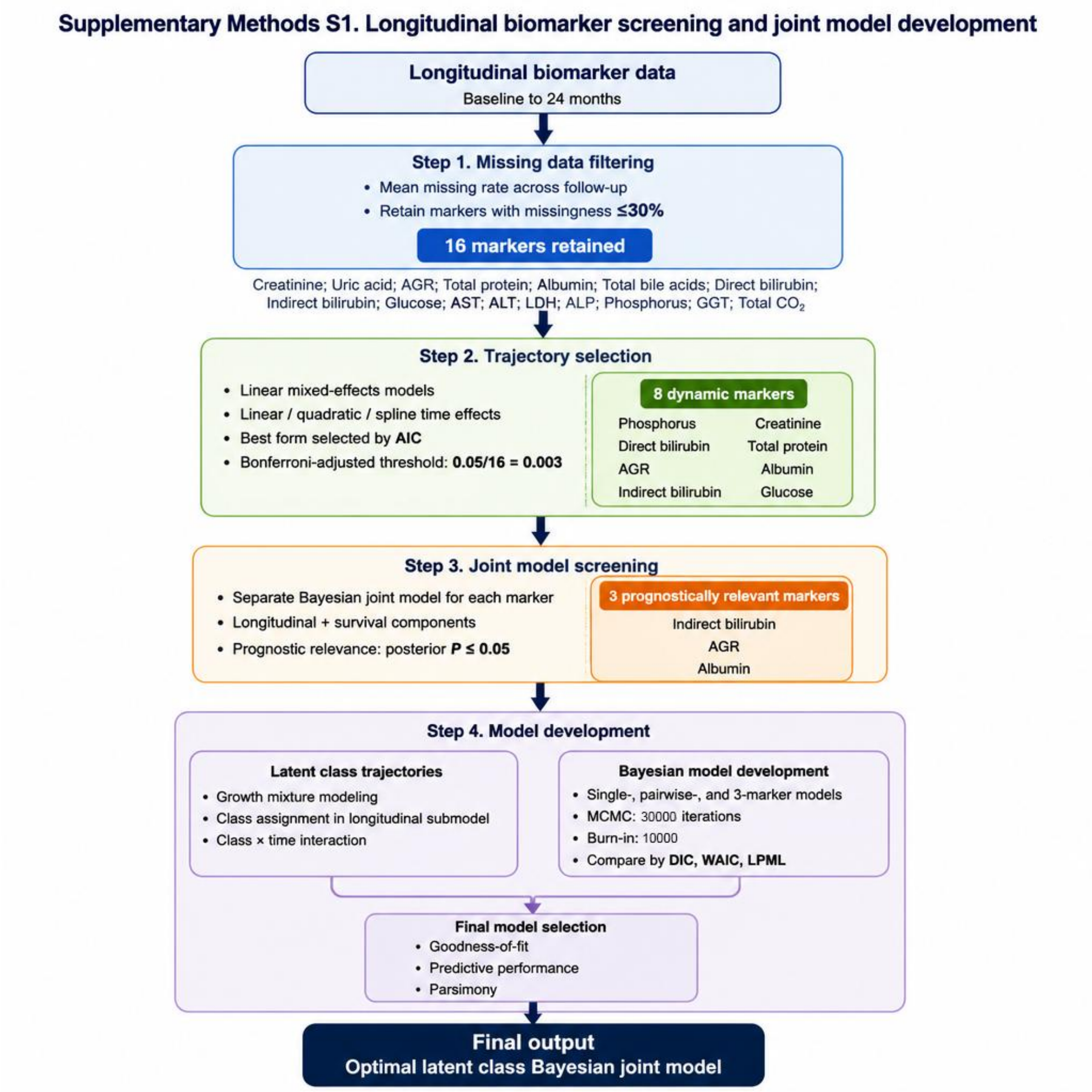

Table S3. Mathematical specification of the Bayesian joint longitudinal–survival model

Model Component	Specification
**Survival Sub-model (Time-to-event)**	hi(t) = h0(t)*exp(γ1*M_stage + γ2*CYRA21-1 + γ3*AST + α*mi(t))
**Longitudinal Sub-model**	yi(t) = mi(t) + εi(t), εi(t) ~ N(0, σ²)<br>mi(t) = β0 + β1*ns(t, 3)*latent_class + b0i + b1i*ns(t, 2), (b0i, b1i) ~ N(0, Σ)

hi(t):Instantaneous event hazard at time t;

$h_0(t)$:Baseline hazard function;

yi(t): observed biomarker value at time t;

mi(t): estimated error-free current value of the underlying subject-specific longitudinal trajectory at time t,

M stage: Metastatic stage (M0/M1);

Cyfra21-1: Serum value of cytokeratin 19 fragment at baseline;

AST: Serum value of aspartate aminotransferase at baseline;

$ns(t, df = k)$:Natural cubic spline of time with k degrees of freedom;

β_0, β_1: Population-level (fixed) coefficients;

b_{0i}: Subject-specific (random) intercept for longitudinal marker;

b_{1i}:Vector of subject−specific random spline coefficients;

$\varepsilon_i(t)$: Within-subject residual error for AGR

α: association parameter linking the estimated error-free current value of the underlying subject-specific longitudinal trajectory to the instantaneous hazard of progression or death

latent_class：AGR Class assignment of each patient derived from the latent class mixed model

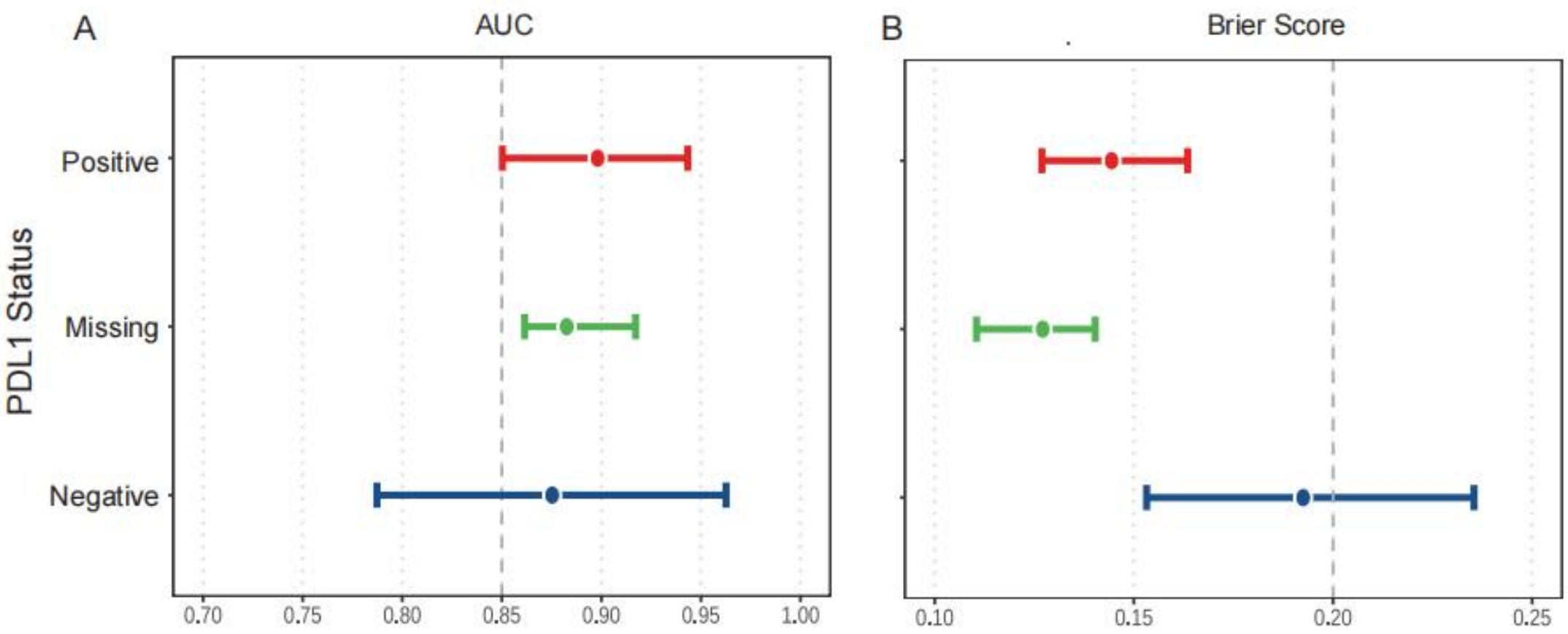

Figure S2. Model performance across PD-L1 subgroups. (**A**) Discriminatory ability assessed by the area under the receiver operating characteristic curve. (**B**) Calibration performance evaluated by the Brier score. The dots represent the mean values, and the horizontal bars indicate the 95% confidence intervals.
